# Defining Rurality: Structural Relationships, Regional Differences, and Applications for Research With Older Adults

**DOI:** 10.1093/geroni/igaa057.2091

**Published:** 2020-12-16

**Authors:** Steven Cohen, Julia McIlmail, Mary Greaney

**Affiliations:** University of Rhode Island, Kingston, Rhode Island, United States

## Abstract

There is no universal definition of rurality due to the heterogeneity in what makes a place “rural” or “urban”. This study explored how elements of rurality are related to each other, and how the elements that define rurality vary by region. Data were abstracted for all 1948 non-metropolitan counties in the contiguous 48 states on rurality. K-means cluster analyses (k=4-8) were conducted to examine classification structures among component variables examining regional differences. In the South region, the majority (51.2%) were “Type 2” counties: low population size and density but higher urbanized population. The Midwest had a majority of “Type 3” counties (56.4%): intermediate for population size and density, but higher distances to metro areas. These exploratory findings underscore the heterogeneity and regional variability in rurality and how those measures are structurally related to each other, and essential to understanding those factors that truly drive rural-urban health disparities for older adults. Part of a symposium sponsored by the Rural Aging Interest Group.

